# Research Highlights, *The Plant Genome*, Volume 15, Issue 4

**DOI:** 10.1002/tpg2.20291

**Published:** 2022-11-22

## 1 TOWARDS NEW VARIETIES OF COCOA TREES THAT ARE LESS BITTER AND MORE AROMATIC

The amount of polyphenols (compounds responsible for bitterness) could be different between modern Nacional varieties and wild trees collected in the ancestral Nacional area. Through the variation in the content of nonvolatile compounds (polyphenols, caffeine, theobromine, proteins, and fat) present in two cocoa (*Theobroma cacao*) populations, Colonges et al. (https://doi.org/10.1002/tpg2.20218) were able to detect areas of the cocoa genome potentially responsible for the variation of nonvolatile compounds in cocoa. They were also able to identify candidate genes whose functions may be involved in the different biosynthetic pathways. These results could provide useful information to define breeding strategies adapted to these traits, as a genomic selection strategy adapted to highly polygenic traits.

## 2 MULTISPECTRAL DATA PREDICT FUTURE BIOMASS

Multispectral data from plants are useful for predicting genotypic and phenotypic values for biomass. The timing for performing multispectral imaging is a critical factor for the prediction accuracy. Sakurai et al. (https://doi.org/10.1002/tpg2.20244) collected time‐series multispectral data from soybean [*Glycine max* (L.) Merr.] in four types of different irrigation levels and evaluated the potential of multispectral data for the prediction of biomass. Sakurai et al. discovered that multispectral data from soybean before flowering stage were useful for predicting the genotypic values of biomass. For phenotypic values of biomass, the optimal timing for multispectral imaging depended on irrigation levels.

## 3 ANOTHER NEW CLUBROOT RESISTANCE GENE

Clubroot, caused by the obligate pathogen *Plasmodiophora brassicae*, causes significant losses in quality and yield of brassica crops worldwide. Additional genes for clubroot resistance in canola (*Brassica napus*) are urgently required because many new pathotypes have been identified from the Canadian Prairies that can overcome the genetic resistance currently available. Rahaman et al. (https://doi.org/10.1002/tpg2.20245) examined a genomic region containing genes involved in resistance to clubroot in vegetable turnip (*B. rapa*) line ECD02 using genetic and genomic approaches. They identified and mapped a gene that provided strong resistance against several of the new pathotypes and developed tightly linked molecular markers. This novel gene, together with other resistance genes that have recently been identified by this research team, will be used by plant breeders to develop resistant cultivars for management of clubroot.

## 4 A SINGLE LARGE EFFECT LOCUS LEADS TO MARKER EFFECT HETEROGENEITY

Genomic prediction relies on assumptions of constant allele substitution effects in the training and prediction population. Rooney et al. (https://doi.org/10.1002/tpg2.20247) found that variation in a single large effect gene, *HvAlaAT* (*Qsd1*), in the *Seed Dormancy* 1 region of chromosome 5H leads to heterogeneous allele substitution effects between groups of otherwise related individuals for germination traits. Heterogenous marker effects led to reduced prediction accuracy when models were trained and predicting on individuals carrying both alleles of *Qsd1* or trained on individuals with one allele and predicting individuals with the opposite allele. Modest gains in prediction accuracy were observed when using a structured genomic best linear unbiased prediction model when the *Qsd1* allele was used to stratify the population. Single large effect loci can lead to violations of the assumptions of standard genomic prediction models.

## 5 WHEAT GENES CONTROLLING PRE‐HARVEST SPROUTING

Preharvest sprout (PHS) occurs when late season rains cause cereal grains to germinate prior to harvest and reduces grain yield and quality. There is a large genetic component to PHS susceptibility with the *TaMFT‐A* gene having been shown to influence PHS. Here, Vetch et al. (https://doi.org/10.1002/tpg2.20250) demonstrate that the *TaMFT‐A* homeologue, *TaMFT‐B2*, has a similar level of effect upon PHS susceptibility. Field experiments were performed on winter wheat (*Triticum aestivum* L.) lines varying for alleles of these genes. The results indicate that functional *TaMFT‐A* and *TaMFT‐B2* promote seed dormancy and reduce PHS with each controlling 14% of PHS variation. *TaMKK3‐4A* and *TaVP1‐3B* were previously associated with control over seed dormancy, however no associations between allelic variation in these genes and PHS were detected in these experiments.

## 6 TOMATO LATE BLIGHT RESISTANCE IDENTIFIED

Late blight, caused by *Phytophthora infestans* (Mont.) de Bary, is one of the most destructive diseases of the cultivated tomato (*Solanum lycopersicum*) worldwide. Genetic changes have resulted in the emergence of new genotypes in the causal pathogen, overcoming formerly effective fungicides or host resistance genes. In this study, Sullenberger et al. (https://doi.org/10.1002/tpg2.20251) characterized the genetic basis of late blight resistance in a new wild variety of tomato. We developed a new molecular genetic map of tomato and used it to identify four genomic locations in the wild species controlling late blight resistance. The results of this study can be used to develop new tomato cultivars with resistance to late blight.

## 7 NOVEL LOCUS FACILITATES WHEAT IMPROVEMENT

Favorable genetic loci and genes for flag leaf‐related traits from wheat (*Triticum aestivum* L.)‐related species have been seldom identified aiming at breaking the current bottleneck of genetic resources in hexaploid wheat. Wang et al. (https://doi.org/10.1002/tpg2.20252) report that loci for flag leaf‐related traits were identified using a tetraploid wheat population of recombinant inbred lines and a genetic map constructed based on a wheat 55K single‐nucleotide polymorphism array. A novel and major interval including loci for flag leaf length, for flag leaf width, for flag leaf area, and for the flag leaf length/width ratio was identified. Based on the genotypes of the closely linked Kompetitive allele‐specific polymerase chain reaction marker, this interval was successfully verified in two F3 populations with different genetic backgrounds. Four genes in this interval were likely involved in the growth and development of the flag leaf size. The detected loci and developed marker will be useful in breeding wheat variety with ideal plant structure.

## 8 SPARSE KERNEL MODELS PROVIDE OPTIMIZATION OF TRAINING SET DESIGN FOR GENOMIC PREDICTION IN WHEAT DATA

Genomic selection (GS) in breeding schemes relies on its ability to provide accurate predictions of unobserved lines at early stages of the breeding program. Multigeneration (multiyear) increases the training data size and improves prediction accuracy. Multigeneration data usually are heterogeneous with complex family relationship patterns. Under these conditions, historical data may not be optimal for model training as the accuracy could be compromised. The sparse selection index (SSI) is a method for training set (TRN) optimization, in which training individuals provide predictions to some but not all predicted subjects. In this study Lopez‐Cruz et al. (https://doi.org/10.1002/tpg2.20254) added an additional trimming process to the original SSI (trimmed SSI) to remove less important training individuals for prediction. Using a large multigeneration (8 yr) wheat (*Triticum aestivum* L.) grain yield dataset (including 68,836 spring wheat lines), we found increases in accuracy as more years are included in the TRN, with small improvements of the trimmed SSI over the traditional Genomic Best Linear Unbiased Predictor (GBLUP).

## 9 MODELING SPATIOTEMPORAL RYEGRASS PHENOTYPES

Accounting for longitudinal measurements of perennial ryegrass (*Lolium* L.) traits can uncover relevant information for breeders and improve the accuracy of genomic prediction models. Bornhofen et al. (https://doi.org/10.1002/tpg2.20255) evaluated random regression models for inference and prediction using multienvironment and multiharvest ryegrass data. The authors report that genetic parameters vary considerably across combinations of environments (spatial dimension) and harvest events (temporal dimension). Genotype × environment interaction was higher in early harvests, whilst genetic variance and heritability increased throughout the growth season. Besides flexibility in different prediction settings, random regression models seem suitable for forecasting future harvests.

## 10 COMPARISON OF GENOTYPE IMPUTATION STRATEGIES

Imputation is a common strategy to obtain high‐density marker data from individuals genotyped with cheaper but lower density chips. In the last years, several imputation tools have been developed for different types of data. Niehoff et al. (https://doi.org/10.1002/tpg2.20257) compared the performance of Beagle, a tool from human genetics, with AlphaPlantImpute2, which was developed for plants. They compare both tools in a rage of different scenarios. The imputation accuracy achieved with Beagle was much lower than that of AlphaPlantImpute2 but could be tremendously improved by optimizing input parameters. Beagle was better in phasing. The best imputation quality was achieved when combining Beagle with AlphaPlantImpute2.

## 11 MULTITRAIT GENOMIC PREDICTION IMPROVES SELECTION ACCURACY FOR MINERAL CONCENTRATIONS IN PEA

Plant breeders are continually working to develop new varieties of nutritious and high yielding peas for farmers to grow and grocers to sell. Because nutritional traits are controlled by numerous genes, plant breeders have developed statistical modeling approaches to predict genetic merit of breeding lines and germplasm that will provide nutritional trait improvement. In this study, Atanda et al. (https://doi.org/10.1002/tpg2.20260) tested multitrait genomic selection models and extended the use of sparse phenotyping method to improve selection accuracy of seed mineral concentrations by maximizing the use of information across related genotypes and genetically correlated traits. Our results suggest the use of heritability and genetic correlation between traits as metrics to further improve prediction accuracy and reduce the cost of phenotyping and time‐consuming data collection process. Our results identified new approaches that should enable breeders to more effectively and efficiently tap diversity and make selection for enhancing seed mineral concentrations and release more nutritious pea varieties to farmers and ultimately consumers.

## 12 IDENTIFICATION OF BROCCOLI AQUAPORINS

Nicolás‐Espinosa and Carvajal (https://doi.org/10.1002/tpg2.20262) have identified the sequence of 65 aquaporin (membrane water transporters) genes in broccoli (*Brassica oleracea* L. var. *botrytis*). The predictive analyses have allowed to determine the functionality of all aquaporin isoforms according to the selectivity to transport water along with other small organic compounds. Also, the study of expression revealed the great importance of the study of aquaporins in broccoli for determining the resistance to environmental stresses.

## 13 GENOMIC SELECTION IMPROVES ALFALFA DROUGHT TOLERANCE

Alfalfa (*Medicago* L.) selection for stress‐prone regions has high priority for sustainable crop–livestock systems. Annicchiarico et al. (https://doi.org/10.1002/tpg2.20264) observed large genotype × environment interaction (i.e., inconsistent biomass yield response of plant material) across drought‐prone environments of Algeria, Morocco, Argentina, and Italy, which emphasizes the need to breed specific cultivars for each region. They compared different genomic selection procedures and reported for best procedures a fairly modest ability to predict the breeding values of plant material in specific environments. However, their results encourage the adoption of genomic selection, when considering the challenges associated with phenotypic selection for stress environments of this outbred crop.

## 14 GENOMIC PREDICTION FOR MAIZE DIVERSITY

The corn (*Zea mays* L.) crop in the United States has limited genetic diversity, potentially rendering it vulnerable to emerging diseases and climate change. Rogers et al. (https://doi.org/10.1002/tpg2.20267) demonstrated that genomic prediction models trained on historical data from the Germplasm Enhancement of Maize project can identify new breeding lines with large genetic contributions from exotic parents that also have superior yield ability.

## 15 YIELD QTL IDENTIFIED IN SOYBEAN

Improving seed yield is one of the main goals of soybean breeding. Ayalew et al. (https://doi.org/10.1002/tpg2.20268) identified consistent quantitative trait loci (QTL) for seed yield in a diversity panel of 541 soybean [*Glycine max* (L.) Merr.] genotypes in Maturity Groups III, IV, and V evaluated in Kansas. Two stable seed yield QTL on chromosomes 9 and 17 were detected in at least three out of four environments. The candidate genes associated with these QTL warrant further analysis to determine their functional mechanisms and develop markers for seed yield improvement.

## 16 GBPR10.5D1 CONTRIBUTES TO WILT RESISTANCE

Verticillium wilt (*Verticillium dahliae* Kleb.) is a severe vascular disease that causes huge yield and economic losses in a wide variety of plants, especially cotton (*Gossypium hirsutum*). Guo et al. (https://doi.org/10.1002/tpg2.20271) report that a pathogenesis‐related protein, GbPR10.5D1, was induced and associated with cotton resistance upon the Verticillium infection. Suppression of GbPR10.5D1 almost abolished the tolerance, whereas overexpression of it in cotton largely improved the resistance. Further analyses indicated GbPR10.5D1 possibly strengthen structural defense and activate defense signaling which released the repression of cell growth caused by the infection. The study explained the underlying mechanism for the resistance of PR10s and provided the possibility for the application of PR10s in molecular breeding.

